# Application of an innovative pancreaticojejunostomy technique with a modified set of perioperative management in pancreatoduodenectomy: a retrospective cohort study

**DOI:** 10.1007/s13304-023-01651-z

**Published:** 2023-10-10

**Authors:** Shiyin Chen, Cheng Zhang, Haifeng Huang, Bin Xi, Jian Zhang, Yibing Jin, Shunliang Gao, Yun Zhang

**Affiliations:** 1 Department of Hepatobiliary and Pancreatic Surgery, Zhejiang Provincial Key Laboratory of Pancreatic Disease, The First Affiliated Hospital, Zhejiang University School of Medicine, Hangzhou, 310003 China; 2 Department of Hepatobiliary and Pancreatic Surgery, Shengzhou Branch Hospital of the First Affiliated Hospital, Zhejiang University School of Medicine, Shengzhou, 312400 China; 3 Department of Hepatobiliary and Pancreatic Surgery, The First Affiliated Hospital, Zhejiang University School of Medicine, Hangzhou, 310003 China

**Keywords:** Pancreaticojejunostomy, Perioperative management, Pancreatic fistula, Postoperative complications, Pancreaticoduodenectomy

## Abstract

How to reduce grade C postoperative pancreatic fistula (POPF) incidence after pancreaticoduodenectomy (PD) is the pursuit of pancreatic surgeons. This study introduced an innovative pancreaticojejunostomy (PJ) technique with a complete set of perioperative management. All 144 patients in this single-center retrospective cohort study underwent the same PJ technique and perioperative management. The primary endpoint was grade C POPF incidence. The secondary endpoints were grade B POPF rate, drain fluid amylase level, complications, hospital stay duration, and mortality. Risk factors for clinically-relevant POPF (CR-POPF) were assessed by logistic regression analysis. No patient (0.0%) experienced grade C POPF, while 44 (30.6%) developed grade B. No in-hospital death was recorded. Multivariate analysis found relatively high body mass index, laparoscopic surgery, and soft or moderate pancreatic texture independent risk factors for CR-POPF. Our novel PJ anastomosis with modified perioperative management helped avoid grade C POPF. However, grade B POPF incidence was relatively high to some extent because of the enhanced management itself.

**Supplementary Information:**

The online version contains supplementary material available at 10.1007/s13304-023-01651-z.

## Introduction

The International Study Group for Pancreatic Fistula defined postoperative pancreatic fistula (POPF) as a common and dire complication after pancreaticoduodenectomy (PD), a commonly performed technique in pancreatic surgery [1]. Although many new improvements have been made in the surgical technique of PD, the POPF incidence remains high, with a biochemical leak (BL) of 7.0–13.9%, grade B POPF rate of 16.4–38.9%, and grade C POPF rate of 2.1–4.6% [2–5]. According to the 2016 update of the International Study Group of Pancreatic Surgery (ISGPS) definition and grading of POPF [1], grades B and C POPF were defined as clinically relevant POPF (CR-POPF) which caused several clinical problems. For instance, intraperitoneal abscess and hemorrhage formation and severe systemic complications such as sepsis and organ failure necessitate catheter drainage or reoperation, resulting in complications and a great economic burden [2, 3, 6]. Grade C POPF had a poor prognosis and led to prolonged postoperative length of stay, high total hospital cost, and even death [7]. Therefore, preventing CR-POPF, especially grade C POPF is currently a very important and challenging clinical issue.

For decades, numerous studies indicated that preoperative risk assessment, surgical technique selection, and perioperative management play critical roles in preventing CR-POPF. The most common risk factors associated with CR-POPF include soft gland texture, high body mass index (BMI), ampullary, duodenal, cystic, or islet cell pathology, small-diameter pancreatic duct, massive intraoperative blood loss, blood transfusion, and longer operative time [8, 9]. Pancreaticojejunostomy (PJ) based on conventional single-loop, double-loop, or modified single-loop reconstruction, and pancreaticogastrostomy (PG) are commonly performed reconstruction approaches after PD [6]. Many anastomotic techniques have been proposed in recent decades, including Cattell–Warren duct-to-mucosa anastomosis [4], Blumgart anastomosis (BA) [10], several modified Blumgart anastomoses (MBA) [10], the Kakita method [11], and invagination anastomosis [6, 12]. External and internal pancreatic ductal stents are widely used for PJ in PD [13]. Besides, the utility of intraperitoneal drainage and somatostatin analogs such as subcutaneous pasireotide in preventing CR-POPF during perioperative management remains debated [14, 15]. A recent systematic review and meta-analysis showed that pancreatic duct occlusion(PDO) had a protective tendency of reducing the risk of grade C POPF after PD compared to pancreatic anastomosis [16]. While some progress has been made in less invasive PD, including laparoscopic and robotic PD [17, 18], it is uncertain whether minimally invasive surgery impacts the occurrence of CR-POPF. Whether open or minimally invasive, there is indeed a need for optimal anastomosis and reasonable perioperative management, especially for high-risk patients.

However, a simple, practical, safe, and reliable management scheme after PD is still absent [19]. Based on clinical practice, we proposed a novel perioperative management and a new anastomotic technique for PJ. We also analyzed the possible value and shortcomings of our method.

## Methods

### Study design

This was a single-center retrospective cohort study. The research subjects were patients who underwent PD at the First Affiliated Hospital of Zhejiang University between June 2013 and June 2020. The primary endpoint of the study was grade C POPF morbidity. The secondary endpoints, assessed over the same interval, included grade B POPF morbidity, drain fluid amylase level, hospital stay duration, in-hospital mortality, and postoperative complications such as delayed gastric emptying (DGE) and post pancreatectomy hemorrhage (PPH). The patients were divided into two groups based on the POPF classification, the no CR-POPF group that included patients with no pancreatic fistula and those with BL, and the CR-POPF group that included grades B and C POPF. Risk factors for CR-POPF were analyzed by comparing the groups for perioperative-related indicators. All surgeries were performed by the same team using the same, along with the same set of perioperative management.

The study included 144 patients. The clinical data collected included age, sex, BMI, comorbidities, surgical history, preoperative biliary drainage history, neoadjuvant therapy history, pancreatic texture, main pancreatic duct diameter, surgical method, intraoperative pancreatic duct expansion, surgical time, hypercoagulability, intraoperative blood loss, hospital stay duration, drain fluid amylase level, POPF, in-hospital mortality related with surgery, DGE, PPH, infection, lymphatic leakage, biliary fistula, organ failure, and management.

The complication classifications were based on the Clavien–Dindo surgical complication classification published in 2009 [20]. Grade III and above were considered serious complications.

### Surgical procedure: an innovative PJ technique

**Fig. 1:**
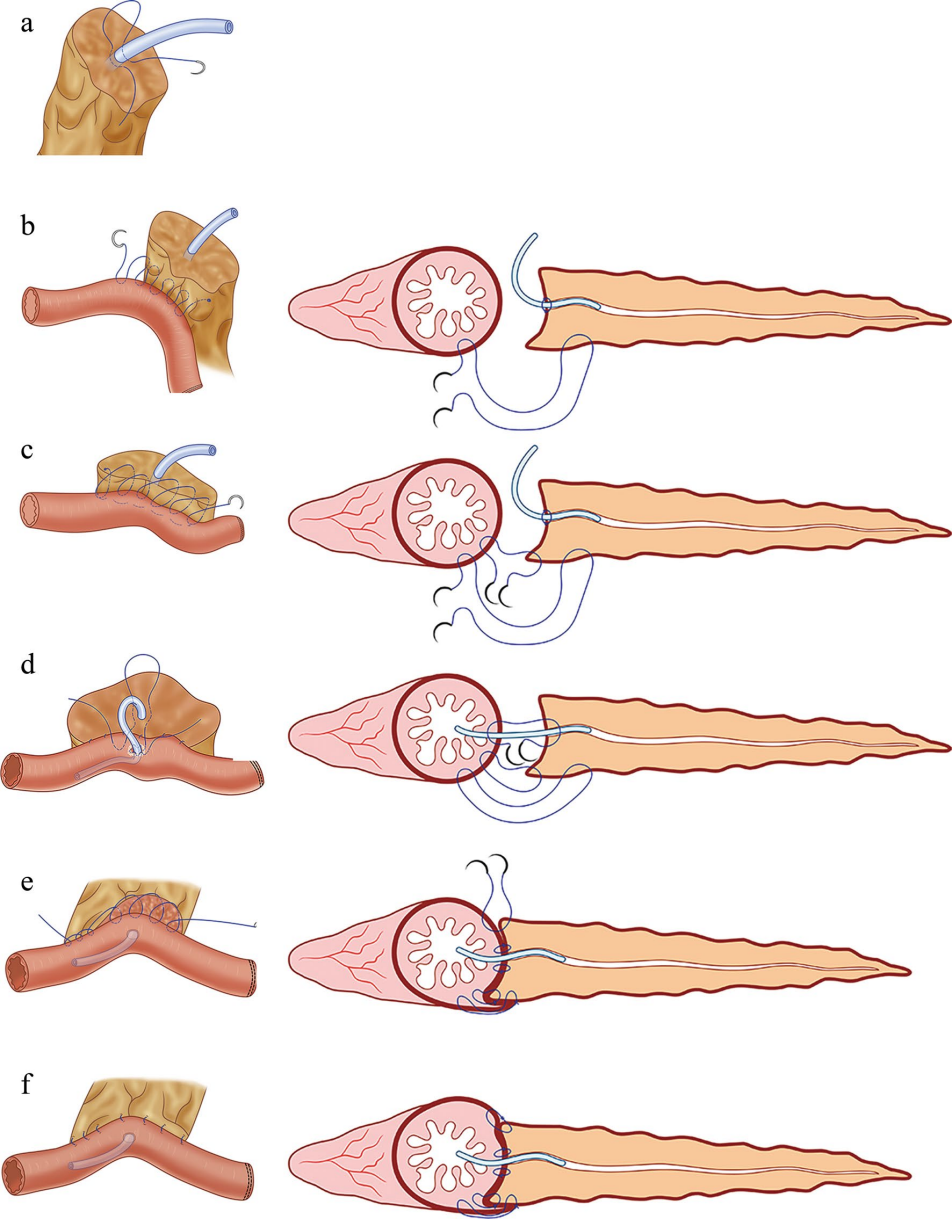
Reinforced dorsal pancreas and double purse-string duct-to-mucosa pancreaticojejunostomy. **a** Place a pancreatic stent inside the main pancreatic duct, and fix it with a purse-string suture. **b** Perform a single-layer continuous suture of the dorsal pancreas and the anterior wall of the jejunum stump near the mesangial margin, 1 cm from the posterior edge of the pancreatic stump. **c** Place a second single-layer continuous suture 0.5 cm from the first layer. **d** Tighten the preset purse-string suture to embed the stump after inserting the stent. **e** Perform a double-layer suture similar to (**c**) to join the ventral pancreas and the jejunum seromuscular layer. **f** Wrap the pancreatic stump

Open or laparoscopic PD was selected based on the patient’s condition and examination results. We adopted our modified duct-to-mucosa PJ method in the surgery. A length of 1–2 cm of the pancreatic stump was freed, and a pancreatic stent of appropriate size was placed in the main pancreatic duct and fixed with a purse-string suture (Fig. 1a). A single-layer continuous suture linking the dorsal pancreas and anterior wall of the jejunum stump near the mesangial margin was performed 1 cm from the posterior edge of the pancreatic stump (Fig. 1b). Another single-layer continuous suture affixed the posterior margin of the pancreatic stump to the jejunum seromuscular layer 0.5 cm from the first layer (Fig. 1c). A small hole at a diameter similar to the pancreatic duct stent was opened in the midpoint of the contralateral mesangial margin of the jejunum and a purse-string suture was placed around it. After directing the other end of the stent to the choledochojejunostomy, we tightened the purse-string suture to embed the stump (Fig. 1d). A double-layer suturing of the ventral pancreas to the jejunum seromuscular layer was similarly performed, one layer 1 cm from the anterior pancreatic stump edge and the other near the anterior edge (Fig. 1e). Finally, the transected pancreatic remnant surface was wrapped (Fig. 1f).

### Perioperative management of CR-POPF

#### First step: intraoperative drainage tube preset

**Fig. 2:**
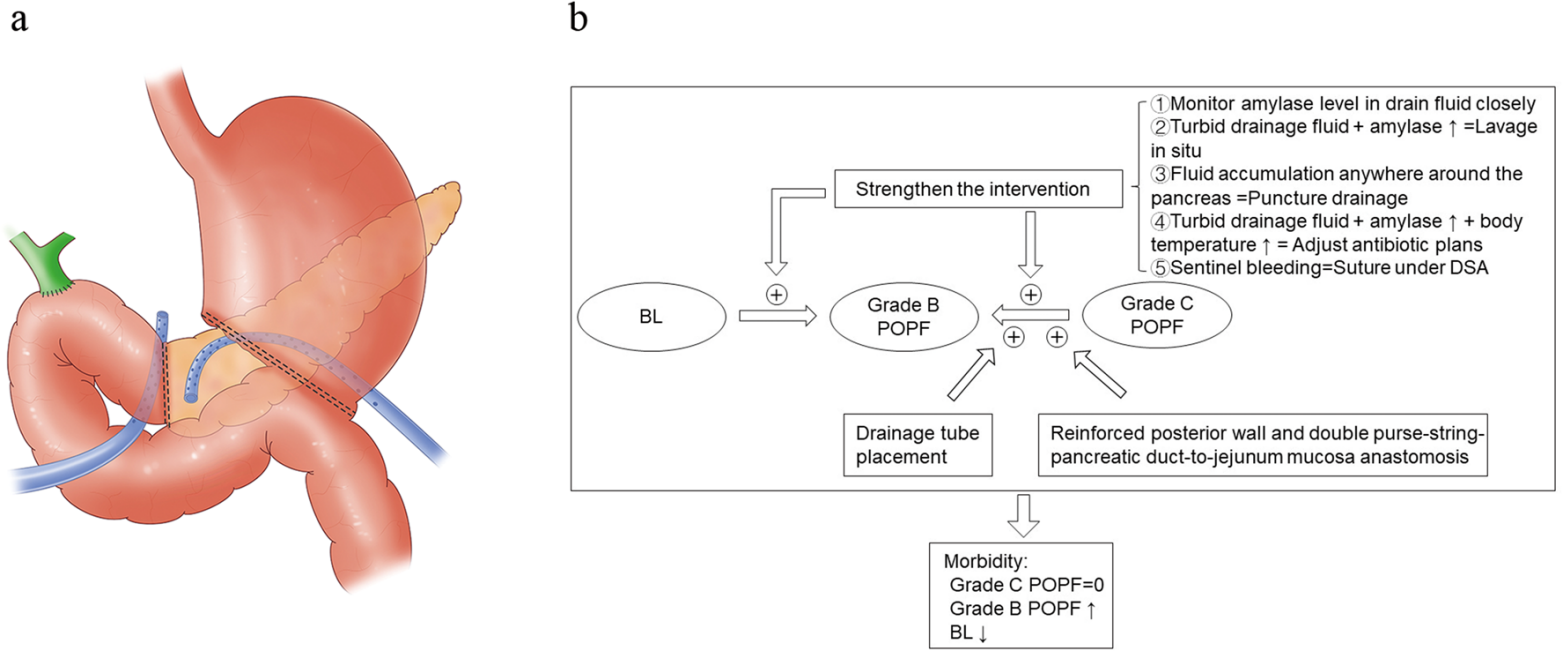
Perioperative management. **a** Drainage tube placement. **b** Strengthen the intervention. *POPF* postoperative pancreatic fistula, *PJ* pancreaticojejunostomy

Two drainage tubes were placed after PJ: one behind the pancreatic-intestinal anastomosis that passed through the choledochojejunostomy to drain potential leaking fluid; the other started in front of the pancreatic-intestinal anastomosis and passed behind the gastrointestinal anastomosis (Fig. 2a).

#### Second step: postoperative monitoring and management

1. The fluid around the anastomosis was routinely tested from the drainage tubes for amylase level and bacteriological smear and culture.
2. In situ lavage was immediately performed if the drainage fluid amylase level increased to over three times higher than the upper limit of the normal serum level, and the drained fluid was turbid or positive during bacterial culture. The so-called in situ lavage meant a large amount of 0.9% saline entered the abdominal cavity at a controlled flow rate through a thin tube inserted into the drainage tube and then flowed out through the thick drainage tube.
3. The computed tomography (CT) scan was conducted as a routine imaging evaluation to find the effusion around the anastomosis that could not be effectively drained, especially behind the anastomosis. Once a distinct effusion was detected, puncture drainage was actively performed to drain the effusion and prevent infection.
4. Bacteriological or empirical antibiotic regulation was performed if the POPF was highly suspected to be associated with infection in case of elevated body temperature, abnormal indicators of infection, or positive drainage culture.
5. If the drainage fluid suddenly turned into blood, we would exclude the anastomotic bleeding under endoscopy, find bleeding vessels by digital subtraction angiography (DSA), and even adopt embolization or coated stent for hemostasis (Fig. 2b).
6. Abdominal exploration for hemostasis would be conducted when bleeding was rapid, or other methods were ineffective.
7. Patients who developed multiple organ failure (MOF) due to complications such as infection and bleeding secondary to POPF were transferred to the intensive care unit (ICU) for organ function support treatment.

According to the 2016 update of ISGPS definition [1]:

1. BL: The drainage fluid amylase level increased to over three times higher than the upper limit of the normal serum level.
2. Grade B POPF: patients who met one of the conditions 2–5.
3. Grade C POPF: patient who met condition 6 or 7.

According to the subclassification of grade B POPF [2]:

1. B1 POPF: patients who experienced persistent drainage (beyond 21 days), without any other treatment.
2. B2 POPF: patients received pharmacological agents such as antibiotics, artificial nutrition (either enteral or parenteral), somatostatin analogs, or blood transfusions for the treatment of the fistula, with or without prolonged drainage.
3. B3 POPF: patients undergoing any interventional procedure short of a reoperation under general anesthesia.

### Statistical analysis

Non-normally distributed continuous variables are presented as median and interquartile range and independent groups were compared using the Mann–Whitney *U* test. Normally distributed continuous variables are presented at mean ± standard deviation and were compared by *t* test. The missing value was replaced by the average value. Categorical variables are described as numbers and percentages and were analyzed using the chi-squared test or Fisher’s exact test, as applicable. Multivariate analysis was performed. Variables perceived to have clinical relevance and resulted in *P* < 0.10 in the univariate analysis were considered candidates for the stepwise binary logistic regression models. The association between CR-POPF morbidity and the predictors are expressed as odds ratios (ORs) and 95% confidence intervals (CIs). The receiver operating characteristic curve analysis was used to identify clinically relevant cut-off values. The diagnostic accuracy was determined based on the area under the curve. The goodness of fit of the binary model was examined using the Hosmer–Lemeshow test. All tests were two-sided, and the significance level was set to 0.05. Data were analyzed using IBM SPSS Statistics for Windows, Version 22.0 (IBM Corp., Armonk, NY, USA).

### Ethics statement

This study was carried out in accordance with the provisions of the Declaration of Helsinki. Institutional review board certification from the First Affiliated Hospital of Zhejiang University was obtained for this study (approval number: 2022-039).

## Results

### Basic clinical data

The study included 144 patients who underwent PJ after PD, aged 62.0 ± 12.4 (range, 21–90) years. Among them, 89 (61.8%) were male.

### Clinical outcomes

#### Primary endpoint

No case of grade C POPF was detected in the 144 patients (0.0%; Table 1).

**Table 1:** Classification and incidence of POPF following the ISGPS 2016 definitions and the subclassification of grade B POPF

POPF grade	Patients included, *n* (%)
None	52 (36.1)
BL	48 (33.3)
Grade B POPF	44 (30.6)
B1 POPF	13 (9.0)
B2 POPF	4 (2.8)
B3 POPF	27 (18.8)
Grade C POPF	0 (0.0)

*POPF* postoperative pancreatic fistula, *ISGPS* International Study Group on Pancreatic Surgery, *BL* biochemical leak

#### Secondary endpoints

Grade B POPF was detected in 44 (30.6%) patients, 48 (33.3%) experienced BL, and 52 (36.1%) did not have pancreatic fistula. Thirteen patients had grade B1 POPF (9.0%), 4 had grade B2 POPF (2.8%), and 27 had grade B3 POPF (18.8%) (Table 1). No patient died during the postoperative course. Compared to the no CR-POPF group, the CR-POPF group had significantly longer hospital stay (22 [15–43] vs. 15 [10–20] days, *P* < 0.001), higher infection rate (47.7% vs. 8.0%, *P* < 0.001), higher Clavien–Dindo grade ≥ III morbidity rate (43.2% vs. 14.0%, *P* < 0.001), and higher drain fluid amylase level (17,172 [3942–44,984] vs. 273 [30–2370] U/L, *P* < 0.001; Table S1).

Since the diagnostic criteria of grade B POPF were relatively subjective, most of them were the expression of the personal will of the physician in charge. In this group of patients, since the treatment of various postoperative conditions had been set up, we could decompose its constituent factors: there were 18 cases of perianastomotic fluid puncture catheterization under ultrasound guidance, 24 cases of lavage due to obvious turbidity in appearance or/and positive bacteriological culture (15 cases) on the basis of significantly increased amylase in drainage fluid, 12 cases of antibiotic adjustment due to abdominal infection secondary to POPF, and 18 cases of delayed removal of drainage tube. The abdominal bleeding caused by POPF was successfully stopped by DSA in 2 cases and by endoscope in 1 case. In 44 patients with grade B POPF, a total of 90 grounds were generated to support grade B POPF, with an average of 2.0 grounds per patient.

#### Analysis of risk factors for CR-POPF

The groups were similar in age, sex, comorbidities, smoking history, alcohol drinking history, pathological types of benign or malignant tumors, surgery history, preoperative biliary drainage history, neoadjuvant therapy history, surgical time, intraoperative blood loss, and hypercoagulability (*P* > 0.05 for all). Compared to the no CR-POPF group, the CR-POPF group had significantly higher rates of patients with BMI > 22.9 kg/m^2^ (59.1% vs. 40.0%, *P* = 0.034) and patients with ampulla of Vater or duodenal tumors (59.1% vs. 35.0%, *P* = 0.007). The pancreatic texture differed significantly between the groups. The gland texture in the no CR-POPF group was soft in 10.0%, moderate in 16.0%, and firm in 74.0% of the patients. The respective values in the CR-POPF group were 31.8, 40.9, and 27.3% (*P* < 0.001). The main pancreatic duct diameter in the CR-POPF group was significantly smaller than in the no CR-POPF group (2 [2, 3] vs. 3 [2–4] mm, *P* = 0.003). The rate of patients with a pancreatic duct diameter ≤ 3 mm in the CR-POPF group was significantly higher than in the no CR-POPF group (88.6% vs. 71.0%, *P* = 0.022). The rate of laparoscopic pancreaticoduodenectomy (LPD) in the CR-POPF group was significantly higher than in the no CR-POPF group (45.5% vs. 20%, *P* = 0.002). Intraoperative pancreatic duct expansion differed significantly between the groups. In the no CR-POPF group, 27.0% of the patients underwent no expansion, 47.0% underwent mild expansion, and 26.0% underwent considerable expansion. The respective values in the CR-POPF group were 52.3, 38.6, and 9.1% (*P* = 0.006; Table S1).

Based on our clinical experience and univariate analysis results, we selected the following potential risk factors for CR-POPF to be included in the binary logistic regression analysis: BMI, LPD or open pancreaticoduodenectomy (OPD), main pancreatic duct diameter ≤ 3 mm, pancreatic duct expansion, and pancreatic texture.

The student residuals of four observations (number 52, 74, 117, 137) were greater than two times the standard deviation, but these were retained in the analysis. The resulting logistic model was statistically significant (*χ*^2^ = 50.287, *P* < 0.001). The model correctly classified 78.5% of the subjects. The sensitivity, specificity, positive predictive value, and negative predictive value of the model were 59.1, 87.0, 66.7, and 82.3%, respectively. Three independent variables included in the model were significant risk factors for CR-POPF: BMI > 22.9 kg/m^2^, laparoscopic surgery, and soft or moderate pancreatic texture. Patients with BMI > 22.9 kg/m^2^ had a 3.206 times higher risk of CR-POPF than those with BMI ≤ 22.9 kg/m^2^ (95% CI 1.287–7.986; *P* = 0.012). The risk of CR-POPF in patients undergoing laparoscopic PD was 4.801 times higher than in patients undergoing open PD (95% CI 1.850–12.464; *P* < 0.001). Patients with a soft and moderate gland were respectively 14.124 and 7.762 times more likely to develop CR-POPF than those with a firm gland (95% CI 4.040–49.382 and 2.681–22.472, respectively; *P* < 0.001 for both; Table 2).

**Table 2:** Results of the univariate and multivariate analyses of risk factors for CR-POPF

Variable	Univariate			Multivariate		
	No CR-POPF (*n* = 100)	CR-POPF (*n* = 44)	*P* value	OR	95% CI	*P* value
Age, mean ± SD	61.95 ± 12.40	61.34 ± 11.61	0.782			
Sex (female/male), *n*	37/63	18/26	0.657			
BMI > 22.9 kg/m^2^, *n* (%)	40 (40.0)	26 (59.1)	**0.034**	3.206	1.287–7.986	**0.012**
Surgery (OPD/LPD), *n*	80/20	24/20	**0.002**	4.801	1.850–12.464	** < 0.001**
Main pancreatic duct diameter ≤ 3 mm, *n* (%)	71 (71.0)	39 (88.6)	**0.022**	3.343	0.743–15.048	0.116
Pancreatic duct expansion, *n* (%)			**0.006**			0.877
No	27 (27.0)	23 (52.3)				
Mildly	47 (47.0)	17 (38.6)		0.779	0.298–2.037	0.611
Considerably	26 (26.0)	4 (9.1)		0.800	0.149–4.281	0.794
Pancreatic texture, *n* (%)			** < 0.001**			** < 0.001**
Firm	74 (74.0)	12 (27.3)				
Soft	10 (10.0)	14 (31.8)		14.124	4.040–49.382	** < 0.001**
Moderate	16 (16.0)	18 (40.9)		7.762	2.681–22.472	** < 0.001**
Ampulla of vater tumor/duodenal tumor, *n* (%)	35 (35.0)	26 (59.1)	**0.007**			

*CR-POPF* clinically-relevant postoperative pancreatic fistula, *SD* standard deviation, *OR* odds ratio, *CI* confidence interval, *BMI* body mass index, *OPD* open pancreaticoduodenectomy, *LPD* laparoscopic pancreaticoduodenectomy

Bold values represents the results with P < 0.05, which were statistically significant.

## Discussion

### Risk factors for CR-POPF

This study demonstrated that relatively high BMI, soft or moderate pancreatic texture, and laparoscopic surgery were risk factors for CR-POPF, as previously reported. It suggested that we pay more attention to patients with these risk factors in the perioperative management of prevention and treatment of CR-POPF. A fragile pancreatic texture, as following fatty infiltration, is easy to break when sutured [21]. The surgery is more complicated and delicate under laparoscopy, and it may be associated with the extended surgical time which could lead to more adverse effects, delaying healing from the trauma. A learning curve associated with minimally invasive PD revealed a significant decrease in the CR-POPF rate with the increasing experience [22]. Other factors such as ampulla of Vater or duodenal tumors, pancreatic duct diameter ≤ 3 mm, and intraoperative pancreatic duct expansion might contribute to CR-POPF, as indicated by the univariate analysis; however, these variables were similar in both groups in the binary logistic regression analysis. The pathological pattern like duodenal or ampullary tumors was associated with soft pancreatic texture, a low degree of fibrosis, and laparoscopic operation, suggesting it was not an independent risk factor, and this parameter was eliminated from the latest risk prediction model for pancreatic fistula [23]. Many studies reported a small pancreatic duct diameter as a risk factor for POPF as it was difficult to anastomose [8, 10]. Male, alcohol consumption history, history of cardiovascular disease, and intraoperative bleeding of over 1000 mL were also reported risk factors for POPF [9, 24, 25]. However, none of them proved significantly associated with pancreatic fistula in our study. The small number of cases and the effect of several confounding factors might account for it.

### The innovative PJ technique

In the study, no patient experienced grade C POPF. In our view, the goal of grade C POPF management should be to eliminate it by improved anastomosis or enhanced perioperative monitoring.

We used an innovative anastomosis technique named the reinforced dorsal pancreas and double purse-string duct-to-mucosa PJ in the study. It was easy to popularize, especially to shorten the learning curve of LPD. Several factors were considered when we modified the traditional pancreatic duct-to-mucosa anastomosis, emphasizing posterior pancreatic reinforcement and performing a double purse-string to embed the pancreatic stump.

First, pancreatic fluid is more likely to leak from the posterior side of the pancreatic-intestinal anastomosis and it is more dangerous. However, the main pancreatic duct is anatomically close to the posterior part where there is less pancreatic tissue to block leakage from the posterior anastomosis. Additionally, suturing on the posterior side is relatively difficult, particularly under laparoscopy. Once posterior pancreatic leakage occurs, the pancreatic fluid accumulates behind the pancreatic-intestinal anastomosis and near the superior mesenteric vein, common hepatic artery, and the bilioenteric anastomosis, corroding blood vessels. This accumulated fluid is extremely difficult to drain. Therefore, it is destined to have a poor prognosis and often develops into CR-POPF. Consequently, we performed that the two suture layers, 1.0 and 0.5 cm from the anastomosis cover the pancreatic tissue behind the pancreatic duct with a relatively wide serous membrane of the small intestine, making it difficult for the pancreatic fluid to leak out.

Second, double purse-string duct-to-mucosa anastomosis is simple to learn and conducive to reducing the technical differences between LPD and OPD. Besides, only three stitches were used, reducing the risk of POPF due to suture tear. Furthermore, purse-string tightening could prevent pancreatic leakage effectively.

### Perioperative management and relatively high rate of grade B POPF

Other than surgical procedures, perioperative management contributed to avoiding grade C POPF by routinely placing drainage tubes, performing earlier lavage of drainage areas when detecting high amylase levels, cloudy drainage fluid, or infection, making timely antibiotic adjustments, extending the indwelling time of abdominal drainage tubes, and stopping bleeding by DSA. And this management approach probably accounted for the increased grade B POPF incidence.

Admittedly, our active intervention probably increased the burden for some low-risk patients. Our precautionary clinical intervention undoubtedly prolonged treatment, possibly involved over-treatment, and increased costs for those who could have healed without intervention. However, we believed that our handling was ethical. Limited by current medical development, we could not predict the patients’ clinical outcomes precisely. Given the considerable harm and poor prognosis of grade C POPF, we chose the lesser of the two evils by proactively enhancing our intervention.

While our innovative approach admittedly had a higher rate of grade B POPF, the important point is that the rate of grade C POPF was zero. The subjectivity of grade B POPF definition might play a crucial role in its high rate, given its close association with postoperative management, as indicated in the International Study Group for Pancreatic Fistula classification of pancreatic fistula [1]. Strategies regarding performing lavage, intervention, drainage, or changing therapy plans vary between surgical teams [12, 25, 26]. When a physician was subjectively aggressive and changed the treatment regimen due to some suspicious conditions in patients originally assessed as BL, the incidence of grade B POPF was bound to increase. Our medical team might be in exactly this state. We closely monitored the drainage fluid, amylase level, the patient’s body temperature, and more. Once they met our intervention indications, we intervened. Some physicians, on the other hand, followed a conservative treatment strategy [12, 26]. Conversely, one could say that our timely intervention protected the patients from grade C POPF. The absence of organ failure, death, or reoperation was what we aimed for in our perioperative management. The essence of our new surgical approach and conservative perioperative management was to significantly reduce the incidence of grade C POPF at the price of increasing grade B POPF incidence.

Different from BL and grade C POPF, the diagnostic criteria for grade B POPF are subjective, clinical heterogeneity within this class might be substantial, and its incidence is affected by perioperative management by the surgical team. Encouragingly, the latest study proposed a subclassification of grade B POPF. Grade B POPF was subclassified into 3 categories (B1: persistent drainage > 21 days, B2: pharmacological treatments; B3: interventional procedures short of reoperation), and the clinical and economic impact of grade B1 POPF was significantly lower than that of B2 and B3 POPF [2]. It was also confirmed by a follow-up study [27]. In our study, 9.0% of patients had grade B1 POPF, 2.8% had grade B2 POPF, and 18.8% had grade B3 POPF. It was worth mentioning that 9 of the 27 B3 patients (6.3%) underwent in situ lavage short of any other interventional procedure or receiving pharmacological agents, 11 (7.6%) received pharmacological agents and underwent in situ lavage short of any other interventional procedure, only 7 (4.9%) was performed percutaneous puncture drainage. The purpose of in situ lavage is to reduce the concentration of pancreatic enzymes and remove as much of the infectious material as possible. However, many teams might not do this, so the number of B POPF and B3 POPF increased in our study. Moreover, persistent drainage, therapeutic antibiotics, artificial nutrition, somatostatin analogues, blood transfusions, and percutaneous intervention were the six most common treatment strategies, while the concept of in situ lavage was not mentioned in the examples of B3 POPF. Therefore, the subclassification of B POPF needs further discussion. We believe that the significance of grading POPF in clinical practice is judging the severity of the condition and providing a treatment basis for clinicians rather than just making a diagnosis after taking measures.

### Limitations

Our study had several limitations. Firstly, it was a retrospective cohort study with limited sample size, and there was no comparison with a referent group. Besides, our patients were heterogeneous in their primary disease, BMI, pancreatic texture, and PD surgical technique (laparoscopic or open). Therefore, a multicenter RCT with a large sample is needed to be conducted in the future, which will divide the patients into the innovative PJ group and the traditional PJ group. Secondly, grade B POPF incidence is relatively high in this study. We will explore a set of more accurate perioperative management in clinical practice in the future, and establish a prediction model for grade C POPF. On the premise of avoiding grade C POPF as much as possible, the clinical intervention of patients should be more precise.

## Conclusions

Our innovative PJ technique with modified perioperative management might help avoid grade C POPF but led to the relatively high incidence of grade B POPF resulting from the active intervention. BMI > 22.9 kg/m^2^, laparoscopic surgery, and soft or moderate pancreatic texture were risk factors for CR-POPF.

### Supplementary Information

Below is the link to the electronic supplementary material.

Supplementary file1 (DOCX 29 KB)

## Data Availability

All data are original from our study. No data from public or shared database was utilized. All materials were originally gained in the First Affiliated Hospital of Zhejiang University.

## Declarations

### Provenance and peer review

Not commissioned, externally peer-reviewed.
