# Supplementary material for: Application of an innovative pancreaticojejunostomy technique with a modified set of perioperative management in pancreatoduodenectomy: a retrospective cohort study
Source: Updates Surg. 2023 Oct 10;75(8):2169–78. doi: 10.1007/s13304-023-01651-z (PMC10710385; doi:10.1007/s13304-023-01651-z)
Supplement: Supplementary file 1 — Supplementary file1 (DOCX 29 KB) [file 13304_2023_1651_MOESM1_ESM.docx]

**Table S1**. Demographic information and perioperative parameters of patients with and without CR-POPF

| Parameter | Total  (*n* = 144) | No CR-POPF (*n* = 100) | CR-POPF  (*n* = 44) | *P*-value |
| --- | --- | --- | --- | --- |

| Age, y, mean ± SD | 61.8 ± 12.1 | 62.0 ± 12.4 | 61.3 ± 11.6 | 0.782 |
| --- | --- | --- | --- | --- |
| Sex (male/female), *n* | 89/55 | 63/37 | 26/18 | 0.657 |
| BMI, kg/m^2^, mean ± SD | 22.9 ± 3.1 | 22.7 ± 3.3 | 23.3 ± 2.6 | 0.224 |
| BMI >22.9 kg/m^2^, *n* (%) | 66 (45.8) | 40 (40.0) | 26 (59.1) | **0.034** |
| Ampulla of Vater tumor/Duodenal tumor, *n* (%) | 61 (42.4) | 35 (35.0) | 26 (59.1) | **0.007** |
| Pathological type, *n* (%) |  |  |  | **0.001** |
| Malignant tumor | 106 (73.6) | 73 (73.0) | 33 (82.5) |  |
| Pancreas | 53 (36.8) | 44 (44.0) | 9 (20.5) |  |
| Bile duct/gallbladder | 26 (18.1) | 13 (13.0) | 13 (29.5) |  |

| Ampulla of Vater/Duodenal | 24 (16.7) | 15 (15.0) | 9 (20.5) |  |
| --- | --- | --- | --- | --- |
| Gastric cancer metastasis | 2 (1.4) | 1 (1.0） | 1 (2.3) |  |
| Retroperitoneum | 1 (0.7) | 0 (0.0) | 1 (2.3) |  |
| Benign tumor | 38 (26.4) | 27 (27.0) | 11 (25.0) |  |
| Pancreas | 25 (17.4) | 19 (19.0) | 6 (13.6) |  |
| Bile duct/gallbladder | 2 (1.4) | 1 (1.0) | 1 (2.3) |  |
| Ampulla of Vater/Duodenal | 9 (6.3) | 6 (6.0) | 3 (6.8) |  |
| Stomach or intestine | 2 (1.4) | 1 (1.0) | 1 (2.3) |  |
| Diabetes mellitus, *n* (%) | 25 (17.4) | 20 (20.0) | 5 (11.4) | 0.208 |
| Hypertension, *n* (%) | 48 (33.3) | 32 (32.0) | 16 (36.4) | 0.609 |
| Smoke, *n* (%) | 39 (27.1) | 27 (27.0) | 12 (27.3) | 0.973 |
| Alcohol consumption, *n* (%) | 25 (17.4) | 14 (14.0) | 11 (25.0) | 0.108 |
| Hyperlipidemia, *n* (%) | 3 (2.1) | 3 (3.0) | 0 (0.0) | 0.246 |
| Pancreatitis, *n* (%) | 2 (1.4) | 0 (0.0) | 2 (4.5) | 0.169 |
| Digestive tract ulcer, *n* (%) | 2 (1.4) | 2 (2.0) | 0 (0.0) | 0.864 |
| Hepatitis, *n* (%) | 4 (2.8) | 2 (2.0) | 2 (4.5) | 0.760 |
| Gastritis, *n* (%) | 2 (1.4) | 2 (2.0) | 0 (0.0) | 0.864 |
| Cerebral infarction, *n* (%) | 6 (4.2) | 3 (3.0) | 3 (6.8) | 0.546 |
| Warfarin, *n* (%) | 1 (0.7) | 1 (1.0) | 0 (0.0) | >0.999 |
| Benign/malignant tumor | 38/106 | 27/73 | 11/33 | 0.802 |
| Previous surgery history, *n* (%) | 39 (27.1) | 25 (25.0) | 14 (31.8) | 0.396 |
| Preoperative biliary drainage history, *n* (%) | 33 (22.9) | 22 (22.0) | 11 (25.0) | 0.693 |
| Neoadjuvant therapy history, *n* (%) | 5 (3.5) | 4 (4.0) | 1 (2.3) | 0.978 |
| Texture of pancreas, *n* (%) |  |  |  | **< 0.001** |
| Firm | 86 (59.7) | 74 (74.0) | 12 (27.3) |  |
| Soft | 24 (16.7) | 10 (10.0) | 14 (31.8) |  |
| Moderate | 34 (23.6) | 16 (16.0) | 18 (40.9) |  |
| Main pancreatic duct diameter, mm, Median (IQR) | 3 (2-3) | 3 (2-4) | 2 (2-3) | **0.003** |
| Main pancreatic duct diameter ≤3 mm, *n* (%) | 110 (76.4) | 71 (71.0) | 39 (88.6) | **0.022** |
| Surgery (OPD/LPD), *n* (%) | 104/40 | 80/20 | 24/20 | **0.002** |
| Intraoperative pancreatic duct expansion, *n* (%) |  |  |  | **0.006** |
| No | 50 (34.7) | 27 (27.0) | 23 (52.3) |  |
| Mild | 64 (44.4) | 47 (47.0) | 17 (38.6) |  |
| Considerable | 30 (20.8) | 26 (26.0) | 4 (9.1) |  |
| Surgical time, min, mean ± SD | 387.21 ± 107.39 | 381.75 ± 110.63 | 396.62 ± 99.76 | 0.360 |
| Infection, *n* (%) | 29 (20.1) | 8 (8.0) | 21 (47.7) | **< 0.001** |
| Hypercoagulability, *n* (%) | 84 (58.3) | 61 (61.0) | 23 (52.3) | 0.328 |
| Blood loss, mL, Median (IQR) | 250 (100–350) | 225 (100–388) | 250 (113–338) | 0.880 |
| Hospital stay duration, d, Median (IQR) | 17 (12–25) | 15 (10–20) | 22 (15–43) | **< 0.001** |
| Drain fluid amylase level, U/L, Median (IQR) | 1 039 (64–14 553) | 273 (30–2 370) | 17 172 (3 942–44 984) | **< 0.001** |
| Clavien-Dindo grade, *n* (%) |  |  |  | **< 0.001** |
| No | 41 (28.5) | 38 (38.0) | 3 (6.8) |  |
| I | 38 (26.4) | 32 (32.0) | 6 (13.6) |  |
| II | 32 (22.2) | 16 (16.0) | 16 (36.4) |  |
| III-a | 22 (15.3) | 9 (9.0) | 13 (29.5) |  |
| III-b | 3 (2.1) | 1 (1.0) | 2 (4.5) |  |
| IV-a | 3 (2.1) | 0 (0.0) | 3 (6.8） |  |
| IV-b | 3 (2.1) | 2 (2.0) | 1 (2.3) |  |
| V | 2 (1.4) | 2 (2.0) | 0 (0.0) |  |
| Clavien-Dindo grade ≥III, *n* (%) | 33 (22.9) | 14 (14.0) | 19 (43.2) | **< 0.001** |
| Mortality, *n* (%) | 0 (0.0) | 0 (0.0) | 0 (0.0) | NA |
| Lymphatic leakage, *n* (%) | 61 (42.4) | 37 (37.0) | 24 (54.5) | 0.050 |
| Biliary fistula, *n* (%) | 5 (3.5) | 2 (2.0) | 3 (6.8) | 0.167 |
| Superior mesenteric vein stenosis, *n* (%) | 1 (0.7) | 0 (0.0) | 1 (2.3) | 0.306 |
| Gastroparesis, *n* (%) | 1 (0.7) | 1 (1.0) | 0 (0) | >0.999 |
| Delayed gastric emptying, *n* (%) | 4 (2.8) | 2 (2.0) | 2 (4.5) | 0.586 |
| Diarrhea, *n* (%) | 13 (9.0) | 10 (10.0) | 3 (6.8) | 0.755 |

| Pneumonia, *n* (%) | 15 (10.4) | 9 (9.0) | 6 (13.6) | 0.391 |
| --- | --- | --- | --- | --- |
| Gastrointestinal and intestinal anastomotic fistula, *n* (%) | 5 (3.5) | 2 (2.0) | 3 (6.8) | 0.167 |
| Heart failure, *n* (%) | 2 (1.4) | 0 (0) | 2 (4.5) | 0.092 |
| Peripancreatic effusion, *n* (%) | 1 (0.7) | 0 (0) | 1 (2.3) | 0.306 |
| Pleural effusion, *n* (%) | 7 (4.9) | 4 (4.0) | 3 (6.8) | 0.437 |
| Urinary tract infection, *n* (%) | 2 (1.4) | 2 (2.0) | 0 (0.0) | >0.999 |
| Abscess, *n* (%) | 1 (0.7) | 1 (1.0) | 0 (0.0) | >0.999 |
| Abdominal infection, *n* (%) | 17 (11.8) | 5 (5.0) | 12 (27.3) | **< 0.001** |
| Intestinal infection, *n* (%) | 11 (7.6) | 8 (8.0) | 3 (6.8) | >0.999 |
| Biliary tract infection, *n* (%) | 1 (0.7) | 1 (1.0) | 0 (0.0) | >0.999 |
| Incision infection, *n* (%) | 7 (4.9) | 2 (2.0) | 5 (11.4) | **0.028** |
| Septic shock, *n* (%) | 1 (0.7) | 0 (0.0) | 1 (2.3) | 0.306 |
| Sepsis, *n* (%) | 6 (4.2) | 4 (4.0) | 2 (4.5) | >0.999 |
| Subhepatic effusion, *n* (%) | 1 (0.7) | 1 (1.0) | 0 (0.0) | >0.999 |
| Ascites, *n* (%) | 11 (7.6) | 7 (7.0) | 4 (9.1) | 0.736 |
| Pelvic effusion, *n* (%) | 1 (0.7) | 1 (1.0) | 0 (0.0) | >0.999 |
| Multiple organ failure, *n* (%) | 2 (1.4) | 2 (2.0) | 0 (0.0) | >0.999 |
| Renal insufficiency, *n* (%) | 3 (2.1) | 1 (1.0) | 2 (4.5) | 0.221 |
| Respiratory failure, *n* (%) | 1 (0.7) | 1 (1.0) | 0 (0.0) | >0.999 |
| Thrombosis, *n* (%) | 1 (0.7) | 1 (1.0) | 0 (0.0) | >0.999 |
| Intestinal obstruction (%) | 1 (0.7) | 0 (0) | 1 (2.3) | 0.306 |
| Hemorrhage (%) | 11 (7.6) | 6 (6.0) | 5 (11.4) | 0.311 |

Abbreviations: CR-POPF, clinically relevant postoperative pancreatic fistula; SD, standard deviation; BMI, body mass index; IQR, interquartile range; OPD, open pancreaticoduodenectomy; LPD, laparoscopic pancreaticoduodenectomy; NA, not reported.

The drain fluid amylase level in the no CR-POPF group is based on *n* = 99 as one value was missing.
